# Syringeable atorvastatin loaded eugenol enriched PEGylated cubosomes in-situ gel for the intra-pocket treatment of periodontitis: statistical optimization and clinical assessment

**DOI:** 10.1080/10717544.2022.2162159

**Published:** 2023-01-05

**Authors:** Heba Amin Elgendy, Amna M. A. Makky, Yara E. Elakkad, Radwa M. Ismail, Nihal Farid Younes

**Affiliations:** a Department of Pharmaceutics, College of Pharmaceutical Sciences and Drug Manufacturing, Misr University for Science and Technology, Giza, Egypt; b Department of Pharmaceutics and Industrial Pharmacy, Faculty of Pharmacy, Cairo University, Cairo, Egypt; c Department of Oral Medicine, Periodontology and Oral Diagnosis, Faculty of Dentistry, Misr University for Science and Technology, Giza, Egypt

**Keywords:** Intra-pocket delivery, periodontitis, atorvastatin, cubosomes, Box-Behnken design, and clinical assessment

## Abstract

Atorvastatin calcium (ATV) is a well-known anti-hyperlipidemic drug currently being recognized for possessing an anti-inflammatory effect. Introducing it as a novel remedy for periodontitis treatment necessitates developing a syringeable modified delivery system capable of targeting inflammation within the periodontal pockets. Thus, a 3^3^ Box-Behnken design was used to generate eugenol enriched PEGylated cubosomes. Based on the desirability function, the optimized formulation (OEEPC) was selected exhibiting a solubilization efficiency (SE%) of 97.71 ± 0.49%, particle size (PS) of 135.20 ± 1.11 nm, polydispersity index (PDI) of 0.09 ± 0.006, zeta potential (ZP) of −28.30 ± 1.84 mV and showing a sustained drug release over 12 h. It displayed a cubic structure under the transmission electron microscope, furthermore, it was stable upon storage for up to 30 days. Hence, it was loaded into an optimum syringeable in-situ gel (ISG) which displayed the desired periodontal gelation temperature (34 ± 0.70 °C) and an adequate gelation time (46 ± 2.82 sec), it also released approximately 75% of the drug within 72 h. Clinical evaluation of the ISG showed a promising percentage reduction of about 58.33% in probing depth, 90% in the bleeding index, 81.81% in the plaque index, and 70.21% in gingival levels of transforming growth factor–β1. This proved that the formulated syringeable intra-pocket delivery system of ATV is an efficient candidate for diminishing inflammation in periodontitis.

## Graphical Abstract

**Figure:**
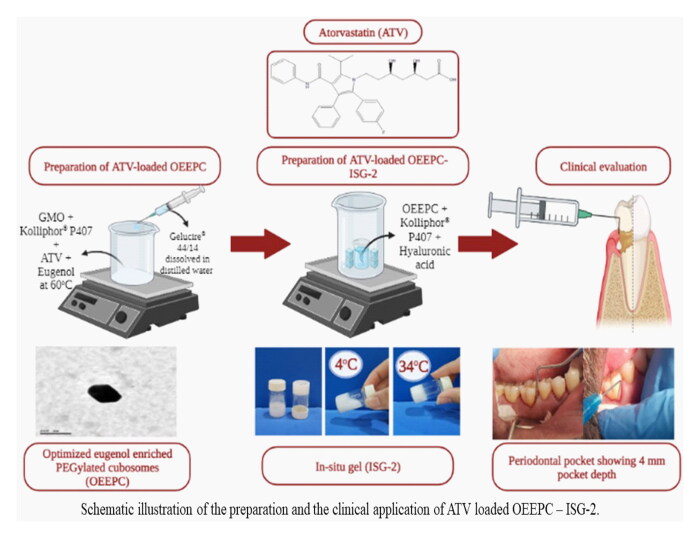

## 1. Introduction

Periodontitis is a destructive chronic inflammatory disease characterized by periodontium damage and pocket formation between the tooth and the gingival margin resulting in gingival retraction and tooth mobility which can eventually cause absolute tooth loss (Shaheen et al., 2020). Consequently, patients with periodontitis often show decreased self-confidence and poor esthetics, which affects their quality of life. Furthermore, periodontitis may enhance the progression of a group of diseases such as diabetes mellitus, atherosclerosis, and chronic obstructive pulmonary disease (Nasra et al., 2017). Accordingly, periodontitis management is a must to maintain oral health and other associated diseases. Removal of plaque and calculus are beneficial in managing periodontitis but are not solely effective after the disease progression (Johnson et al., 2020). So, controlling the inflammation through the administration of either systemic or local anti-inflammatory drugs is considered a necessity in periodontal therapy (Ruan et al., 2016). However, patients favor local drug delivery systems due to their reasonable cost, ease of application, diminished side effects, and rapid onset of action (Johnson et al., 2020). Yet, formulation of these systems remains challenging as they have various limitations such as poor penetration into the subgingival cavity and limited residence time due to the high turnover rate of gingival crevicular fluid (GCF) in periodontal pockets (Hau et al., 2014). This calls for the development of novel intra-pocket drug delivery systems capable of targeting the periodontal pockets with effective drug concentration.

Atorvastatin calcium (ATV) is a common specific competitive inhibitor of 3-hydroxy-2-methyl-glutaryl coenzyme A (HMG-CoA) reductase widely used in the treatment of hyperlipidemia (Dong et al., 2018). There is emerging evidence that ATV possesses several pleiotropic effects such as wound healing, anti-inflammatory, antioxidant, and immunomodulatory effects (Tahamtan et al., 2020). Due to these beneficial effects, various studies investigated the efficacy of ATV as a protective and therapeutic agent for periodontitis and favorable outcomes were attained (Pradeep et al., 2013; Balli et al., 2014; Surve et al., 2015). However, the systemic administration of ATV has limited therapeutic efficacy in the dental route due to its poor oral bioavailability (∼12%) caused by the rapid metabolic degradation and poor aqueous solubility (0.1 mg/ml) (Choudhary et al., 2012). To address these limitations, delivering ATV directly into the periodontal pockets through modified local delivery systems can be a good alternative in periodontitis treatment.

Cubosomes are three-dimensional cubic nanostructures formed by self-assembled amphiphilic lipids upon dispersion in excess aqueous medium proposing a well-defined honeycombed structure (Mansour et al., 2021). However, steric stabilizers are essential due to the lack of colloidal stability. Nonionic surfactants such as Kolliphor^®^ P 407 are mostly preferred due to their ability to preserve the bi-continuous internal structure of cubosomes (Chen et al., 2014). Cubosomes have several remarkable properties due to their unique structure such as biocompatibility, bioadhesiveness, biodegradability, facile inclusion of both hydrophilic and hydrophobic drugs, and ability to control drug release rate (Chen et al., 2014).

Eugenol is a natural phenolic monoterpene found in clove. Previous literature reported that it exerted an analgesic effect in the management of periodontitis through the inhibition of macrophage function controlling the inflammatory reactions in the dental pulp (Jadhav et al., 2004). Moreover, it employed an anti-inflammatory action through the inhibition of the cyclooxygenase-II enzyme and provided pain relief through an anti-nociceptive action (Ahmad et al., 2019). The utilization of eugenol in a concentration of ≤5% is considered safe and adequate for topical application without local irritation (Opdyke, 1975). Its beneficial effects qualify it as an attractive option to be used in dentistry (Kim & Park, 2015).

In-situ gelling systems (ISG) are polymeric solutions that are flowable at ambient temperature and tend to stiffen into a viscous gel upon getting in contact with the delivery site (Okur et al., 2020). Activation of the sol-to-gel transition of these systems can be initiated by various microenvironmental triggers such as temperature, pH, and ionic strength. Recently, thermosensitive gels have come to the attention of drug formulators owing to their flexibility in adjusting the properties of these systems according to the administration route and the required therapeutic effect. Kolliphor^®^ P 407 has been extensively used as an invertible thermo-gelling polymer for being safe and hydrosoluble. Yet, it suffered from poor mechanical properties and rapid erosion that can be countered by the use of mucoadhesive polymers. Remarkably, much recent research has focused on hyaluronic acid (HA) as a mucoadhesive polymer in in-situ gelling systems due to its viscoelasticity, biocompatibility, and biodegradability properties (Liao et al., 2005). Additionally, the utilization of HA in dentistry can be advantageous thanks to its wound healing, bone regeneration, anti-edematous, and anti-inflammatory effects (Eick et al., 2013).

Recently, the demand for the development of a sustained intra-pocket drug delivery system had increased. So, this study aims to formulate an ISG incorporating ATV loaded into cubosomes enriched with eugenol with the aid of Gelucire^®^ 44/14 as a safe PEGylated surfactant to solubilize ATV and enhance its deposition into the periodontal pocket. To achieve this, a 3^3^ Box-Behnken design is used to evaluate and optimize the cubosomal formulations according to the desirability function. Finally, the optimized formulation is loaded into an ISG and characterized by various *in-vitro* studies, and evaluated clinically on systemically healthy patients.

## 2. Materials and methods

### 2.1. Materials

Atorvastatin calcium (ATV) was generously supplied by Misr Company for pharmaceutical industries (Giza, Egypt). Glyceryl monooleate (GMO), Kolliphor^®^ P 407, Eugenol, and cellulose membrane (12,000 – 14,000 molecular weight cutoff) were purchased from Sigma-Aldrich Corp., St. Louis, MO, USA. Gelucire^®^ 44/14 (lauroyl macrogolglycerides) was kindly donated by Gattefossé (Saint-Priest Cedex, France). Hyaluronic acid (HA) was supplied by DSM personal care and beauty (Basel, Switzerland). Ethanol 95% and Methanol were purchased from Al-Nasr Chemical Co. (Cairo, Egypt).

### 2.2. Methods

#### 2.2.1. Preparation of eugenol enriched PEGylated cubosomes (EEPCs)

EEPCs were prepared using the top down-method (Younes et al., 2018b). Briefly, GMO and Kolliphor^®^ P 407 (10% w/w of the total dispersion) were melted together on a hot plate magnetic stirrer (model MSH-20D, GmbH, Germany) at 60 °C with continuous stirring at 1500 rpm forming the lipid phase. Then both ATV (120 mg) and eugenol (2.5% w/w of the lipid phase) was added to the lipid phase. Afterward, the preheated aqueous phase (10 ml) containing Gelucire^®^ 44/14 as the aqueous phase stabilizer in different concentrations (0, 2, and 4%) was injected dropwise into the lipid phase. Afterward, this blend was homogenized using a high-shear homogenizer (Remi Electrotechnik, Vasai, India, RQT 127/A/D) at 10,000 rpm at different time intervals (0, 5, and 10 min) and was left for continuous stirring for 60 min at 1500 rpm to obtain a homogenous dispersion. The formulation was left overnight in the refrigerator (4 °C) before further investigation.

#### 2.2.2. Statistical optimization design

The different EEPCs were prepared according to a 3^3^ Box-Behnken design using Design-Expert^®^ software version 13 (Stat-Ease, Inc., Minneapolis, Minnesota, USA) to analyze the effects of different variables on the preparation of ATV-loaded EEPCs, 15 formulations were prepared. The independent variables were lipid phase concentration (X_1_), Gelucire^®^ 44/14 concentration (X_2_), and homogenization time (X_3_), whereas other formulation variables were all kept constant. The dependent variables studied were solubilization efficiency (SE%), particle size (PS), polydispersity index (PDI), percentage of ATV released after 0.5 h (Q_0.5h_), and percentage of ATV released after 8 h (Q_8h_) (Y_1_, Y_2_, Y_3_, Y_4,_ and Y_5_, respectively). Independent variables investigated with the actual values of their levels are shown in Table 1. The detailed composition of the resulting formulations is shown in Table 2.

**Table 1.** Independent variables with their levels in the 3^3^ Box-Behnken design for EEPCs formulations preparation, model summary statistics, constraints for optimization, the selected optimized levels, and the actual and predicted responses of the optimized EEPCs (OEEPC) with the calculated percent deviation for validating the used models.

Factors (independent variables)	Levels				Optimized levels			
	Low (-1)		Medium (0)	High (+1)				
X_1_: Lipid phase concentration (%)	2.5		7.5	12.5	11.86			
X_2_: Gelucire^®^ 44/14 concentration (%)	0		2	4	3.47			
X_3_: Homogenization time (min)	0		5	10	10			
**Responses (dependent variables)**	**Model summary statistics**				**Constraints**	**Evaluation of optimized formulation**		
	R^2^	Adjusted R^2^	Prediction R^2^	Adequate precision		Actual values	Predicted values	Percent deviation
Y_1_: SE (%)	0.982	0.975	0.957	39.273	Maximize	97.71	97.90	0.19
Y_2_: PS (nm)	0.995	0.989	0.965	47.732	Minimize	135.20	130.01	3.98
Y_3_: PDI	0.975	0.943	0.902	22.553	Minimize	0.09	0.09	9.15
Y_4_: Q_0.5h_ (%)	0.916	0.883	0.858	17.645	Minimize	8.17	8.08	1.06
Y_5_: Q_8h_ (%)	0.964	0.954	0.939	30.211	Maximize	76.99	77.79	1.03

**Abbreviations:** EEPCs, eugenol enriched PEGylated cubosomes; SE%, solubilization efficiency; PS, particle size; PDI, polydispersity index; Q_0.5h_, percent of drug released after 0.5 h, and Q_8h_, percent of drug released after 8 h.

**Table 2.** Experimental runs, independent variables, and measured responses of 3^3^ Box-Behnken design of EEPCs formulations. Data are displayed as mean ± SD, (*n* = 3).

F	Lipid phase concentration (%)	Gelucire^®^ 44/14 concentration (%)	Homogenization time (min)	DC (%)	SE (%)	PS (nm)	PDI	ZP (mV)	Q_0.5h_ (%)	Q_8h_ (%)
F1	2.5	2	10	114.09 ± 1.07	59.02 ± 1.97	140.20 ± 1.20	0.28 ± 0.04	−25.70 ± 0.50	14.50 ± 5.32	91.92 ± 11.07
F2	2.5	0	5	101.75 ± 3.37	45.27 ± 3.68	155.17 ± 3.21	0.27 ± 0.01	−28.20 ± 4.52	17.65 ± 6.51	97.60 ± 2.48
F3	2.5	2	0	114.63 ± 0.84	57.45 ± 0.51	160.00 ± 7.07	0.23 ± 0.004	−25.60 ± 1.06	15.28 ± 5.66	91.50 ± 4.29
F4	2.5	4	5	91.79 ± 0.61	68.48 ± 2.80	148.50 ± 2.82	0.13 ± 0.01	−23.90 ± 3.00	12.93 ± 1.96	80.99 ± 6.35
F5	7.5	0	0	98.99 ± 6.42	57.57 ± 5.79	162.10 ± 0.66	0.24 ± 0.03	−27.50 ± 1.20	15.42 ± 10.01	95.26 ± 1.02
F6	7.5	2	5	102.68 ± 0.62	73.94 ± 0.14	132.30 ± 0.40	0.23 ± 0.006	−27.90 ± 0.70	11.05 ± 4.23	83.96 ± 4.06
F7	7.5	4	10	103.84 ± 0.76	90.28 ± 3.45	106.90 ± 8.48	0.09 ± 0.009	−26.10 ± 1.26	9.52 ± 1.87	78.82 ± 25.58
F8	7.5	4	0	112.63 ± 0.92	82.25 ± 4.23	201.30 ± 2.54	0.33 ± 0.007	−26.20 ± 1.36	10.27 ± 3.55	75.46 ± 17.25
F9	7.5	0	10	109.17 ± 2.81	68.52 ± 5.21	162.00 ± 2.00	0.27 ± 0.006	−27.50 ± 0.76	13.37 ± 3.76	97.96 ± 17.33
F10	7.5	2	5	112.95 ± 1.22	72.99 ± 1.65	139.00 ± 1.55	0.28 ± 0.01	−29.70 ± 1.76	14.11 ± 4.01	87.07 ± 20.65
F11	7.5	2	5	111.97 ± 1.22	76.56 ± 1.93	137.00 ± 1.64	0.24 ± 0.004	−23.80 ± 0.49	14.16 ± 1.31	89.07 ± 4.24
F12	12.5	4	5	108.72 ± 0.61	97.90 ± 0.69	183.30 ± 4.38	0.22 ± 0.01	−27.20 ± 1.59	7.41 ± 2.62	73.85 ± 15.54
F13	12.5	2	0	114.12 ± 0.15	90.95 ± 1.32	213.20 ± 2.82	0.40 ± 0.02	−30.40 ± 0.95	11.18 ± 3.01	84.60 ± 8.84
F14	12.5	2	10	111.54 ± 2.82	91.48 ± 1.16	148.80 ± 10.04	0.15 ± 0.003	−22.80 ± 0.47	9.97 ± 3.18	83.50 ± 4.06
F15	12.5	0	5	100.47 ± 3.15	83.84 ± 2.48	192.20 ± 8.34	0.22 ± 0.009	−28.10 ± 1.65	11.22 ± 1.74	92.52 ± 12.42

**Notes:** The amounts of ATV and eugenol are the same in all formulations. Gelucire^®^ 44/14 concentration is calculated from the aqueous phase.

**Abbreviations:** ATV, atorvastatin calcium; EEPCs, eugenol enriched PEGylated cubosomes; DC%, drug content; SE%, solubilization efficiency; PS, particle size; PDI, polydispersity index; ZP, zeta potential; Q_0.5h_, percent of drug released after 0.5 h, and Q_8h_, percent of drug released after 8 h.

#### 2.2.3. Characterization and optimization of ATV-loaded EEPCs

##### 2.2.3.1. Drug content

To determine the drug content, 0.1 ml of the dispersion was appropriately diluted using methanol. Afterward, the UV absorbance of the diluted sample was determined using a UV spectrophotometer (model UV-1601 PC, Shimadzu, Kyoto, Japan) at λ_max_ 244 nm (Dong et al., 2018). The drug content was measured using the following equation (El-Leithy et al., 2018):

Eq. (1) $$\text{Drug content }(\%)\;=\;\frac{\mathrm{Actual}\;\mathrm{yield}}{\mathrm{Theoritical}\;\mathrm{yield}}\;x\;100$$

##### 2.2.3.2. Solubilization efficiency (SE%)

The solubilized amount of ATV was determined after suitable dilution of the filtered dispersion using methanol. The UV absorbance was determined using a UV spectrophotometer (model UV-1601 PC, Shimadzu, Kyoto, Japan) at λ_max_ 244 nm. The SE% was calculated using the following equation (Younes et al., 2018b):

Eq. (2) $$\text{SE\% }=\;\frac{\text{Weight of solubilized ATV in filtered EEPCs }}{\text{Total ATV content}}\;x\;100$$

##### 2.2.3.3. Particle size (PS), polydispersity index (PDI), and zeta potential (ZP)

The mean PS and PDI of EEPCs were determined using the dynamic light scattering technique at 25 °C using Zetasizer (Malvern Instruments, Malvern, UK). ZP was carried out to determine the surface charge of the particles by measuring their electrophoretic mobility in the electrical field. All samples were appropriately diluted with deionized water before measurements.

##### 2.2.3.4. In-vitro release study

The *in-vitro* release study was performed by using a USP dissolution tester apparatus II (Pharma Test, Hainburg, Germany) to evaluate the rate of release of the drug from different EEPCs formulations. An adequate volume of the dispersions equivalent to 10 mg ATV was placed in an 8 cm long presoaked dialysis bag (12–14 kDa, Sigma-Aldrich, St. Louis, USA) sealed with a clamp to prevent leakage. Then the cellulose membrane was placed in a flask containing 200 ml of distilled water and ethanol (1:1) (Nasra et al., 2017; Bansal et al., 2018). The system was stirred at constant speed at 100 rpm at 37 °C. At predetermined time intervals (0.5, 1, 2, 4, 6, and 8 h), aliquots (3 ml) of the samples were withdrawn and replaced by a fresh release medium to maintain sink condition. The withdrawn aliquots were analyzed using a UV spectrophotometer at λ_max_ 244 nm to determine the amount of ATV released at each time interval.

#### 2.2.4. Optimization and validation

The optimized eugenol enriched PEGylated cubosomes (OEEPC) was selected using Design-Expert^®^ software by minimizing PS, PDI, and Q_0.5h_ while maximizing SE% and Q_8h_. According to the desirability function, the OEEPC was prepared and tested to determine its SE%, PS, PDI, Q_0.5h_, and Q_8h_. The actual values of these responses were compared to the predicted values from the model using the following equation (Younes & Habib, 2022):

Eq. (3) $$\text{Percent}\;\text{deviation}\;\left(\%\right)\;=\;\left(\left|Y_{m\;\;\text{predicted}\;\text{values}}-{\;Y}_{m\;\;\text{actual}\;\text{values}}\right|/\left(Y_{m\;\;\text{predicted}\;\text{values}}\right)\right)\;x\;100$$

#### 2.2.5. Transmission electron microscopy (TEM)

A transmission electron microscope (Joel JEM 1230, Tokyo, Japan) was used to examine the morphological structure of OEEPC. First, appropriate dilution of the sample was performed, and a drop was dried over a carbon-coated copper grid and stained with 2% w/v phosphotungstic acid solution. Subsequently, imaging of the sample was performed using TEM at 80 kV (Albash et al., 2021).

#### 2.2.6. Differential scanning calorimetry (DSC)

The differential scanning calorimeter (DSC-50, Shimadzu, Japan) calibrated with purified indium was used to generate the thermograms of pure ATV, GMO, Kolliphor^®^ P 407, eugenol, Gelucire^®^ 44/14, physical mixture of the OEEPC, and the lyophilized OEEPC. Approximately 5 mg of each sample was sealed in an aluminum pan and heated at a temperature range of 25 °C to 300 °C at a scanning rate of 10 °C/min under a nitrogen purge of 50 ml/min (Younes et al., 2021).

#### 2.2.7. Fourier-transform infrared (FTIR) spectroscopy

The drug-excipient compatibility was determined using FTIR spectroscopy. FTIR-8400 (Shimadzu, Kyoto, Japan) was used to analyze the IR spectra of ATV, GMO, Kolliphor^®^ P 407, eugenol, Gelucire^®^ 44/14, physical mixture of the OEEPC, and the lyophilized OEEPC at ambient temperature. Approximately 2 – 3 mg of each sample was compressed into a disk using dry KBr and then scanned at the scanning range of 400–4000 cm^−1^ (Younes et al., 2021).

#### 2.2.8. Stability study

The OEPPC was stored in tightly closed, amber-colored glass vials at refrigeration temperatures of 4 – 8 °C for 30 days (Risaliti et al., 2019). The stored system was visually inspected for any phase separation or sedimentation. SE%, PS, ZP, PDI, Q_0.5h_, and Q_8h_ were remeasured after 30 days and the results were statistically analyzed using paired t-test using SPSS^®^ software 22.0 (SPSS, Chicago, IL, USA). The release profile of the stored system was compared to that of a freshly prepared one according to the model-independent mathematical approach of Moore and Flanner (1996). The similarity factor (ƒ_2_) was calculated according to the following equation (Al-Mahallawi et al., 2014):

Eq. (4) $${ƒ}_{2}=\;50\;\;\log\;\;\{{\left[1\;+\;(\frac{1}{n})\sum_{\left(t=1\right)}^{n}\;\;{\left(R_{t}{}_{\;}–{\;T}_{t}\right)}^{2}\right]}^{-0.5}x\;100\}$$

where n is the number of sampling points, R_t_ and T_t_ are the mean percent released from the reference (fresh) and from the test (stored) at time t. An ƒ_2_ value ≥ 50 indicates that the release profiles are similar, whereas smaller values may imply dissimilar release profiles.

#### 2.2.9. Preparation of in-situ gel (ISG)

The ISG was prepared by adding Kolliphor^®^ P 407 in different concentrations (9, 9.5, and 10% w/v) to an adequate volume of the OEEPC dispersion equivalent to 1.2% w/v of ATV (Pradeep et al., 2013; Kumari et al., 2017). The mixture was left to stir using a magnetic stirrer (model MSH-20D, GmbH, Germany) at 4 °C using an ice bath for 1 h at 1500 rpm. Then 0.2% w/v HA was added to the previous mixture and left to stir under the same conditions.

#### 2.2.10. Characterization of the in-situ gel (ISG)

##### 2.2.10.1. Gelation temperature

To investigate the temperature at which the dispersion turns to gel, 5 ml of each formulated ISG was continuously stirred using a magnetic stirrer (model MSH-20D, GmbH, Germany) at 30 rpm with a gradual increase of temperature at a constant rate (2 °C/min). The temperature at which the magnet stops is the gelation temperature (Erol et al., 2020).

##### 2.2.10.2. Gelation time

The test tube inversion method was employed to determine the gelation time. 2 ml of the selected ISG (ISG-2) was placed in a test tube, which was immersed in a thermostatic water bath (Yongguangming Medical Instrument Company, Beijing, China) having a temperature equivalent to the gelation temperature of the formulation. The time the dispersion takes to turn into a gel with no signs of flow upon inversion of the test tube was considered the gelation time.

##### 2.2.10.3. Viscosity and rheological behavior measurement

The viscosity and rheological behavior of the selected ISG (ISG-2) was studied using a cone and plate viscometer (Brookfield programmable DVII + Model pro II type, USA). The viscosity of the ISG-2 was assessed at two different temperatures (25 °C and 34 °C) and a constant speed (10 rpm) to determine the effect of temperature change on the ISG (Hamed et al., 2019). The rheological behavior of the ISG-2 at 25 °C and 34 °C was measured at different velocities (10, 20, 30, 40, and 50 rpm) with 10 s between every two successive speeds and then was repeated in descending order of the velocity (Morsi et al., 2016). The rheogram of the ISG was represented and the shear rate (y-axis) was plotted against the shear stress (x-axis). Furthermore, Farrow’s equation was used to assess the rheological behavior of the formulation (Elakkad et al., 2021):

Eq. (5) Farrow’s equation:  Log  G = N  Log  F –  Log  η

where, G is the shear rate (sec^−1^), N is Farrow’s constant, F is shear stress (dynes/cm^2^), and η is viscosity (cP). For shear-thickening systems, N ˂ 1 while for Newtonian systems, N approaches 1, whereas N exceeds 1 in the case of shear thinning systems.

##### 2.2.10.4. Syringeability study

An aliquot of 1 ml of the selected ISG (ISG-2) was placed in a 21-gauge needle syringe and gentle force was applied by pressing the injector part of the syringe. Then, the ease of syringeability of the gel through the needle was determined (Nasra et al., 2017).

##### 2.2.10.5. In-vitro release study of in-situ gel

The release of ATV from the drug suspension (10 mg/ml), OEEPC, OEEPC loaded ISG-2 and ATV loaded ISG-2 was performed using a USP dissolution tester apparatus II (Pharma Test, Hainburg, Germany) as previously mentioned. Aliquots were withdrawn at predetermined time intervals (0.5, 1, 2, 4, 6, 8, 10, 12, 24, 48, and 72 h). A statistical evaluation of the rate of ATV release from different systems was performed using univariate followed by Tukey’s post-hoc test using SPSS^®^ software 22.0 (SPSS, Chicago, IL, USA). Moreover, the release profiles were further analyzed using the DDSolver software (Excel Add-in) by fitting the obtained data into different model equations to evaluate the release mechanism from different systems. The best-fit model was selected based on the highest correlation coefficient (r^2^) (Rapalli et al., 2021; Musallam et al., 2022).

#### 2.2.11. Clinical study design

##### 2.2.11.1. Patients’ grouping

The study was approved by Misr for Science and Technology University Institutional Review Board (MUST-IRB) of research ethics (Approval number: FWA00025577 – December 4, 2021), and the protocol complied with the Declaration of Helsinki (1975, as revised in 2000) for biomedical research involving human subjects and the principles of Good Clinical Practice. Twenty-four systemically healthy patients of both genders with ages ranging from 25 to 45 years old with moderate periodontitis were recruited from the oral diagnosis clinic of the Faculty of Oral and Dental Surgery at Misr University for Science and Technology. Smokers, pregnant and lactating patients, and patients with systemic diseases were excluded from the study. The selected volunteers had no history of periodontal therapy and no history of antibiotic intake for the past six months with no history of allergy to any of the formulation ingredients. After patients’ recruitment and applying the inclusion and exclusion criteria, the informed consent was signed by each patient. A day before starting the study period, patients were randomly allocated to one of the three study groups by applying a computerized generated table for their equal and even distribution into the groups. Group 1: received non-surgical scaling and debridement alone. Group 2: received non-surgical scaling and debridement with ATV loaded OEEPC – ISG-2 at a dose of 1.2% ATV (Pradeep et al., 2013; Kumari et al., 2017). Group 3: received non-surgical scaling and debridement with ATV loaded ISG-2 at a dose of 1.2% ATV (Pradeep et al., 2013; Kumari et al., 2017).

##### 2.2.11.2. Treatment protocol

Scaling and debridement were performed for all the allocated patients. Thirty minutes later, either ATV loaded OEEPC – ISG-2 or ATV loaded ISG-2 was administered into the periodontal pockets according to the previous grouping. ISG application was performed continuously using a syringe with a bent needle that was directly inserted into the periodontal pocket until pocket overfilling was recognized. After day 7, patients were recalled for the re-application of the treatments. After drug application, patients were instructed not to eat or drink for 4 hours and to maintain oral hygiene during the study period. They were also instructed not to take any anti-inflammatory drugs, antibiotics, or anti-plaque medications two weeks before the start of the study until the end of the study period (Gad et al., 2017). On days 14 and 42, patients were recalled only for regular follow-ups to maintain their oral hygiene.

##### 2.2.11.3. Clinical parameters

Clinical parameters including probing depth (PD), bleeding index (BI) and plaque index (PI) were recorded twice in the study; at baseline (before scaling and debridement) and at end of the study (at day 42) (Kapil et al., 2018). To determine the impact of the studied delivery systems on the mean values of the different clinical parameters, a percent reduction was calculated on day 42 using the following equation (Shaheen et al., 2020):

Eq. (7) $$\text{Percent reduction }(\%)\;=\;\frac{\text{Parameter at baseline}-\text{Parameter on day }42}{\text{Parameter at baseline}}\text{ x }100$$

###### 2.2.11.3.1. Probing depth (PD).

 The assessment of PD is a valuable clinical parameter used for the clinical assessment of connective tissue destruction. To determine the PD, the probing pocket per site around the tooth was measured from the gingival margin to the base of the periodontal pocket using a University of North Carolina no. 15 (UNC 15) periodontal probe calibrated in 1-mm intervals (Gad et al., 2017).

###### 2.2.11.3.2. Bleeding index (BI).

 The bleeding on probing was scored as an objective sign of inflammation using the standard probe according to the following criteria; zero = no bleeding on probing, 1 = pinpoint bleeding on probing, 2 = confined line of bleeding on probing, 3 = triangular bleeding at interdental papillae on probing, and 4 = profuse bleeding upon probing (Carter et al., 1974).

###### 2.2.11.3.3. Plaque index (PI).

 It was used to assess plaque accumulation around the gingival margin. The degree of plaque accumulation was assessed according to the following criteria; zero = no plaque in the gingival area, 1 = a film of plaque adhering to the free gingival margin and adjacent area of the tooth, and the plaque was recognized only by running a probe across the root surface, 2 = moderate accumulation of soft deposition within the gingival pocket and on the gingival margin and/or adjacent to the tooth surface, which can be seen by the naked eye, and 3 = abundance plaque accumulation within the gingival pocket and/or on the gingival margin and adjacent to the tooth surface (Gad et al., 2017).

##### 2.2.11.4. Assessment of transforming growth factor–β1 (TGF-β1)

TGF-β1 is an anti-inflammatory cytokine that is produced mainly by regulatory T-cells and macrophages in addition to its beneficial biological effects on gingival and periodontal fibroblasts enhancing their mitosis. Previously, literature reported the efficacy of TGF-β1 as a marker for monitoring the periodontal condition and referring to the degree of inflammation relief (Vikram et al., 2015). The levels of TGF-β1 in gingival crevicular fluid (GCF) were evaluated at different time points (baseline, 7, 14, and 42 days (at the end of the study)). GCF was collected from the sulcus of the deepest pocket for each patient by using paper points size 25 after complete isolation of the tooth. Paper points were placed in the sulcus until mild resistance was sensed and then left in place for 30 seconds. Contaminated paper points with saliva or blood were excluded. After GCF collection, paper points were placed in Eppendorf vials and kept at −80 °C. The levels of TGF-β1 were measured using Enzyme-Linked Immunosorbent Assay (ELISA) kits for TGF-β1 (Boster, USA, lot no.: 2271211217) (Agrali et al., 2016). To determine the impact of the studied delivery systems on the mean TGF-β1 levels, the percent reduction equation was calculated on day 42 (Shaheen et al., 2020).

##### 2.2.11.5. Statistical analysis of the data

Data analysis was performed statistically using SPSS^®^ software 22.0 (SPSS, Chicago, IL, USA). The comparison between each parameter at baseline and the end of the study was conducted using paired t-test. Conversely, the comparison between different groups at different periods was performed using univariate and the post-hoc test was assessed using Tukey test. The significance level was set at *p* < 0.05.

## 3. Results and discussion

### 3.1. Drug content

As shown in Table 2, all the formulations showed a drug content ranging from 91.79 ± 0.61% to 114.12 ± 0.15% lying in the acceptable range set by the British Pharmacopeia (85 - 115%) (British Pharmacopoeia Commission, 2011).

### 3.2. Solubilization efficiency (SE%)

SE% of ATV ranged from 45.27 ± 3.68% to 97.90 ± 0.69% as shown in Table 2. This response showed a linear model with a non-significant lack of fit (*p* = 0.3956). The polynomial model for SE% (Y_1_) was presented by the following equation:

Eq. (8) $$\text{SE}{\%}_{}=\;74.44\;+\;16.74X_{1}+\;10.46X_{2}+\;2.63X_{3}-\;2.29X_{1}X_{2}$$

**Figure 1.**
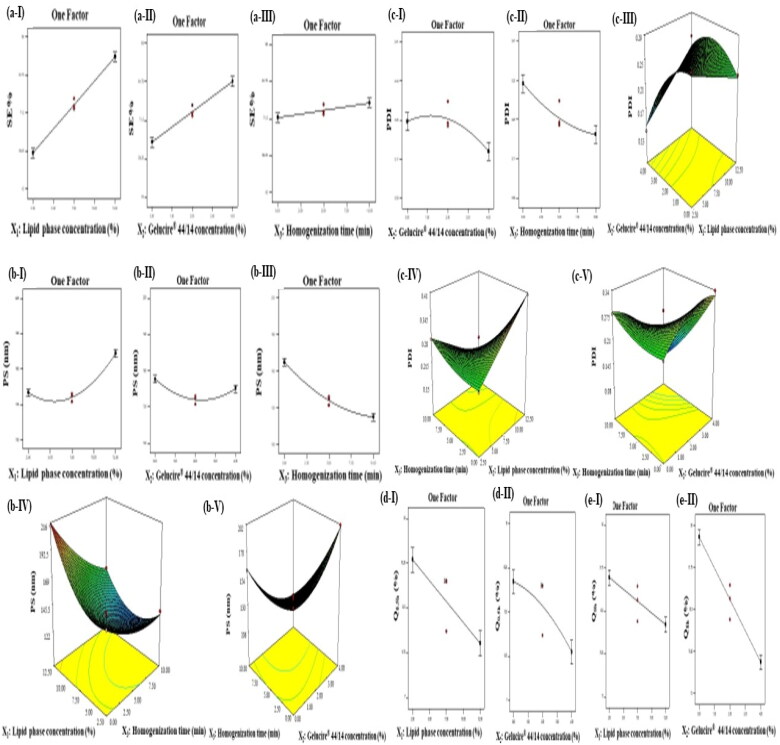
Line plots for the significant effects of lipid phase concentration (X_1_), Gelucire^®^ 44/14 concentration (X_2_), and homogenization time (X_3_) on (a) SE%, (b) PS, (c) PDI, (d) Q_0.5h_ and (e) Q_8h_ together with the response surface plots of the significant interactions.

Lipid phase concentration (X_1_) had a significant positive linear effect on SE% (*p* < 0.0001). Increasing the concentration of the lipid phase, which is composed of GMO, eugenol and Kolliphor^®^ P 407 significantly increased SE% as shown in Figure 1a-I. Owing to the hydrophobic nature of GMO (mono-, di- and triglycerides of fatty acid) and eugenol (LogP = 2.61), the solubilization of the hydrophobic ATV (BCS class II, LogP = 6.98) was significantly increased (Pernin et al., 2019; Shahraeini et al., 2020). Furthermore, Kolliphor^®^ P 407 might have enhanced the solubilization of ATV due to its emulsification effect (Singla et al., 2020).

Gelucire^®^ 44/14 concentration (X_2_) had a significant positive linear effect on SE% (*p* < 0.0001). Increasing this variable significantly increased SE% as shown in Figure 1a-II due to the unique composition of Gelucire^®^ 44/14 (i.e. mono and di-esters of PEG, mono-glycerides, and oily phase (di- and tri-) glycerides), in addition to its high HLB value of 14. Similar results were reported by Karataş et al. who observed the enhancement of the solubilization of the hydrophobic drug piroxicam by the utilization of Gelucire^®^ 44/14 (Karataş et al., 2005).

Homogenization time (X_3_) had a significant positive linear effect on SE% (*p* = 0.0112). Increasing this variable significantly increased SE% as shown in Figure 1a-III due to the increased contact time between the drug and the constituents forming cubosomes in presence of high mechanical shear which enhanced the drug solubilization and accommodation into the cubosomal dispersion (Younes et al., 2018b).

### 3.3. Particle size (PS)

PS of EEPCs ranged from 106.90 ± 8.48 nm to 213.20 ± 2.82 nm as shown in Table 2 indicating that all EEPCs were in the nanometric range. The model fits a reduced quadratic model with a non-significant lack of fit (*p* = 0.7152). The polynomial model for PS (Y_2_) was presented by the following equation:

Eq. (9) $$\text{PS}\;=\;136.10\;+\;16.70X_{1}–\;3.93X_{2}–\;22.34X_{3}–\;11.15X_{1}X_{3}–\;23.58X_{2}X_{3}+\;20.58X_{1}^{2}+\;13.11X_{2}^{2}+\;8.87X_{3}^{2}$$

Lipid phase concentration (X_1_) had a significant positive linear effect (*p* < 0.0001) and a significant positive quadratic effect on PS (*p* < 0.0001). Increasing this variable significantly decreased PS until a certain limit after which PS increased as shown in Figure 1b-I. Increasing GMO might have improved the solubilization of the drug and hence its emulsification forming smaller particles. However, a further increase in the concentration of the lipid phase might have increased the viscosity of the dispersion hindering the division of cubosomes into smaller structures (Nithya et al., 2018).

Gelucire^®^ 44/14 concentration (X_2_) had a significant negative linear effect (*p* = 0.0087) and a significant positive quadratic effect on PS (*p* = 0.0001). Increasing this variable significantly decreased PS until a certain limit after which PS increased as shown in Figure 1b-II. This might be attributed to the adsorption of the Gelucire^®^ 44/14 molecules on the lipid/aqueous interface minimizing the interfacial tension, thus allowing their efficient emulsification, and keeping their size at its minimum. However, a further increase in Gelucire^®^ 44/14 concentration might have elevated the viscosity of the cubosomal dispersion resulting in the fusion of particles and hindrance of the droplets subdivision. Moreover, the increase of Gelucire^®^ 44/14 concentration resulted in its accumulation on the surface of cubosomes increasing the PS (Aboud et al., 2018).

Homogenization time (X_3_) had a significant negative linear effect (*p* < 0.0001) and a significant positive quadratic effect on PS (*p* = 0.0011). Increasing this variable significantly decreased PS (*p* < 0.0001) as shown in Figure 1b-III. This could be attributed to the high mechanical and hydraulic shear which conferred energy to the dispersion leading to the breakage of the cubic structure into smaller structures with low tendency of aggregation (Asadinezhad et al., 2019).

Interaction between lipid phase concentration and homogenization time (X_1_X_3_) had a significant negative effect on PS (*p* = 0.0003) as shown in Figure 1b-IV. While that between Gelucire^®^ 44/14 concentration and homogenization time (X_2_X_3_) had a significant negative effect on PS (*p* < 0.0001) as shown in Figure 1b-IV.

### 3.4. Polydispersity index (PDI)

PDI values provided an insight into the width of the size distribution of the cubosomal dispersion, which ranges from 0 (completely monodisperse particles) to 1 (highly polydisperse particles) (Al-Mahallawi et al., 2014). PDI of EEPCs ranged from 0.09 ± 0.006 to 0.40 ± 0.02 as shown in Table 2. The model fits a reduced quadratic model with a non-significant lack of fit (*p* = 0.9192). The polynomial model for PDI (Y_3_) was presented by the following equation:

  Eq. (10) $$\text{PDI}\;=\;0.25\;+\;9.500E-00{3X}_{1}–\;0.0{31X}_{2}–\;0.0{52X}_{3}+\;0.0{35X}_{1}X_{2}–\;0.0{75X}_{1}X_{3}–\;0.0{71X}_{2}X_{3}–\;0.0{36X}_{2}{}^{2}+\;0.0{19X}_{3}{}^{2}$$

Lipid phase concentration (X_1_) had no significant effect on PDI (*p* = 0.1993). Conversely, Gelucire^®^ 44/14 concentration (X_2_) had a significant negative linear effect (*p* = 0.0035) and a significant negative quadratic effect on PDI (*p* = 0.0100). Increasing this variable significantly increased PDI up to a certain limit after which PDI decreased as shown in Figure 1c-I. The effect of Gelucire^®^ 44/14 concentration on PDI was in contrast with its effect on PS. Increasing its concentration from 0% to 2% might have formed smaller PS but with non-uniform distribution, while the further increase from 2% to 4% formed larger particles with a more uniform distribution.

Homogenization time (X_3_) had a significant negative linear effect (*p* = 0.002) and a non-significant positive quadratic effect on PDI (*p* = 0.1016). Increasing this variable significantly decreased PDI as shown in Figure 1c-II due to the high shear force exerted on the cubosomes which resulted in a homogenous dispersion (Asadinezhad et al., 2019). This result was in parallel to that observed by Younes et al. in which increasing the homogenization time formed a more uniform dispersion (Younes et al., 2018b).

Interaction between lipid phase concentration and Gelucire^®^ 44/14 concentration (X_1_X_2_) had a significant positive effect on PDI (*p* = 0.0091) as shown in Figure 1c-III. Contrarily, the interaction between lipid phase concentration and homogenization time (X_1_X_3_) had a significant negative effect on PDI (*p* = 0.0002) as shown in Figure 1c-IV. Furthermore, the interaction between Gelucire^®^ 44/14 concentration and homogenization time (X_2_X_3_) had a significant negative effect on PDI (*p* = 0.0003) as shown in Figure 1c-V.

### 3.5. Zeta potential (ZP)

The values of the ZP of the EEPCs ranged from −22.80 ± 0.47 mV to −30.40 ± 0.95 mV as shown in Table 2. High negative ZP values indicated high repulsion forces between the cubosomal particles which prevented their aggregation and coalescence and hence maintained their stability. The negative charge on the cubosomes might be intimately related to the oleic acid found in GMO where the ionization and dissociation of its carboxylic group imparted the surface charge (Eldeeb et al., 2019). Additionally, the presence of hydroxyl ions in Kolliphor^®^ P 407 might have augmented the negative charge found on the surface of the cubosomes (Salem et al., 2022).

### 3.6. In-vitro release study

The release of ATV from different EEPCs showed a biphasic pattern with an initial burst followed by a more sustained release of the remaining drug for a period of 12 h. This result runs with that published by Elnaggar et al., who attributed this behavior to the fast release of the drug weakly bound to the surface of cubosomes followed by a slower release of the drug molecule solubilized into the lipid core of the cubosomes (Elnaggar et al., 2015). The percentage of ATV released from different EEPCs after 0.5 h and 8 h were listed in Table 2. The model fits a linear model (*p* < 0.0001) with a non-significant lack of fit (*p* = 0.9941) and (*p* = 0.9341) for Q_0.5h_ and Q_8h_, respectively. The polynomial models for Q_0.5h_ (Y_4_) and Q_8h_ (Y_5_) were presented by the following equations:

Eq. (11) $$Q_{0.5h}=\;12.90\;–\;2.{57X}_{1}–\;2.{19X}_{2}–\;0.60X_{3}–\;0.{67X}_{2}{}^{2}$$

Eq. (12) $$Q_{8h}=\;86.94\;–\;3.{44X}_{1}–\;9.{28X}_{2}+\;0.{67X}_{3}$$

Lipid phase concentration (X_1_) had a significant negative linear effect on both Q_0.5h_ and Q_8h_ (*p* < 0.0001). Increasing this variable significantly decreased the amount of drug released after 0.5 h and 8 h as shown in Figure 1d-I and e-I, respectively. This might be related to the increased viscosity of the dispersion which hindered the release of the lipophilic drug from the cubic structure to the release medium (Eldeeb et al., 2019).

Gelucire^®^ 44/14 concentration (X_2_) had a significant negative linear effect on both Q_0.5h_ and Q_8h_ (*p* < 0.0001) and a non-significant negative quadratic effect on Q_0.5h_ (*p* = 0.1978). Increasing this variable significantly decreased the amount of drug released after 0.5 h and 8 h as shown in Figure 1d-II & e-II, respectively. This could be attributed to the coating of the cubosomes by the surfactant molecules which might have increased the distance through which the drug diffuses resulting in a diminished drug release rate. This result is in accordance with that previously reported by Hegazy et al. (Hegazy et al., 2022). On the other hand, the homogenization time (X_3_) had no significant effect on both Q_0.5h_ and Q_8h_ (p > 0.05).

### 3.7. Optimization and validation

For optimization, Design expert^®^ software suggested optimized eugenol enriched PEGylated cubosomes (OEEPC) formulation of maximum desirability function (0.864) composed of lipid phase concentration (11.86%), Gelucire^®^ 44/14 concentration (3.47%) and homogenization time (10 min). As shown in Table 1, the percent deviation (%) calculated for all dependent variables were found to be less than 10%. All the model analyses exhibited rationale agreement between the adjusted R^2^ and predicted R^2^ and precision values greater than 4 indicating the sufficiency of the models to navigate the space with the high signal-to-noise ratio of the results and confirming the validity of the final selected models (Younes et al., 2018a).

### 3.8. Transmission electron microscopy (TEM)

**Figure 2.**
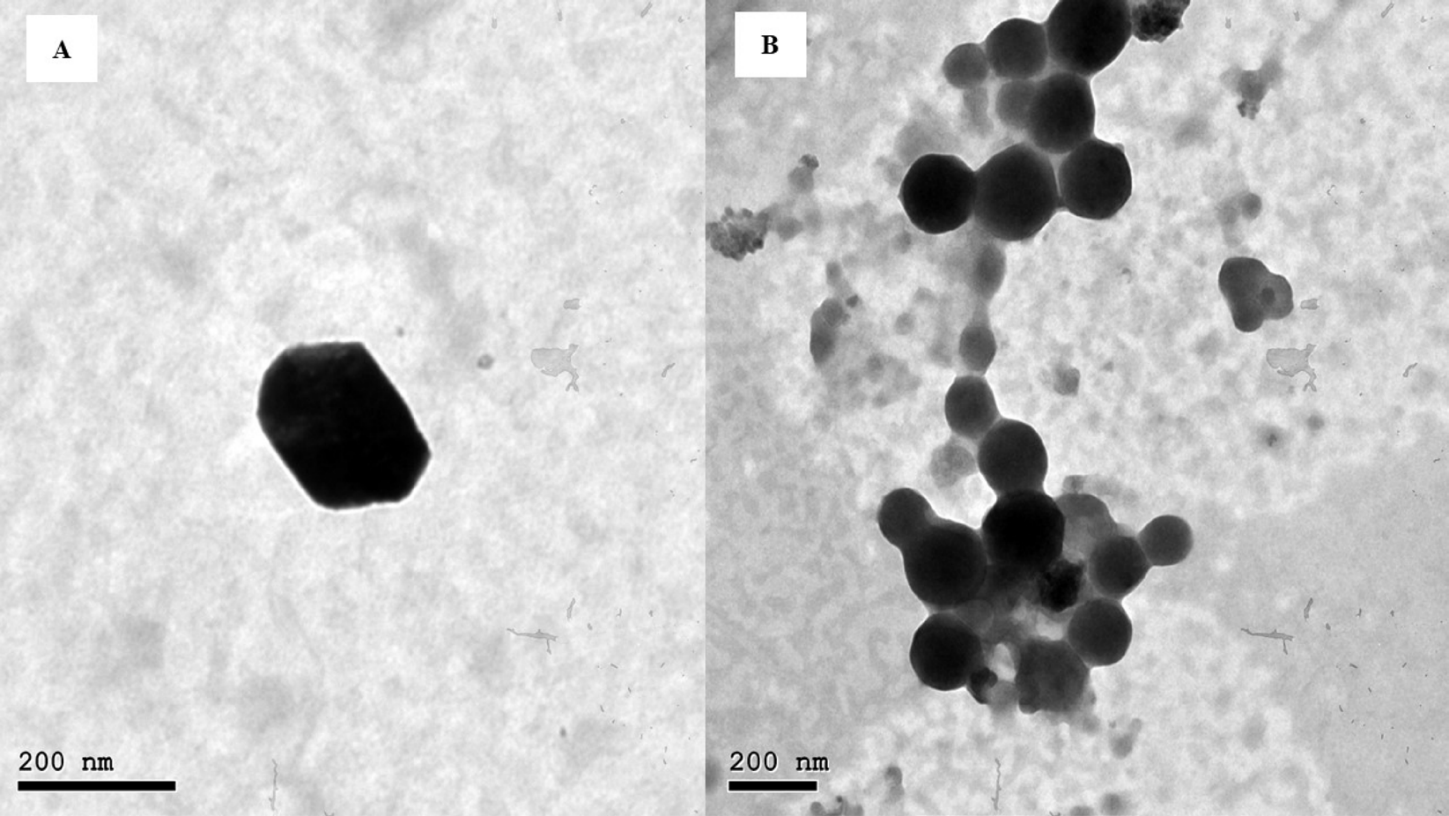
Transmission electron micrographs of the optimized formulation (OEEPC).

As presented in Figure 2, micrographs of OEEPC using the high magnification power of TEM, revealed a defined cubic structure with a smooth surface. The images confirmed that the drug molecules were well solubilized into the cubic dispersion, moreover, the optimized formulation was in the nanometric range which was in accordance with the measurements of the Zetasizer.

### 3.9. Differential scanning calorimetry (DSC)

**Figure 3.**
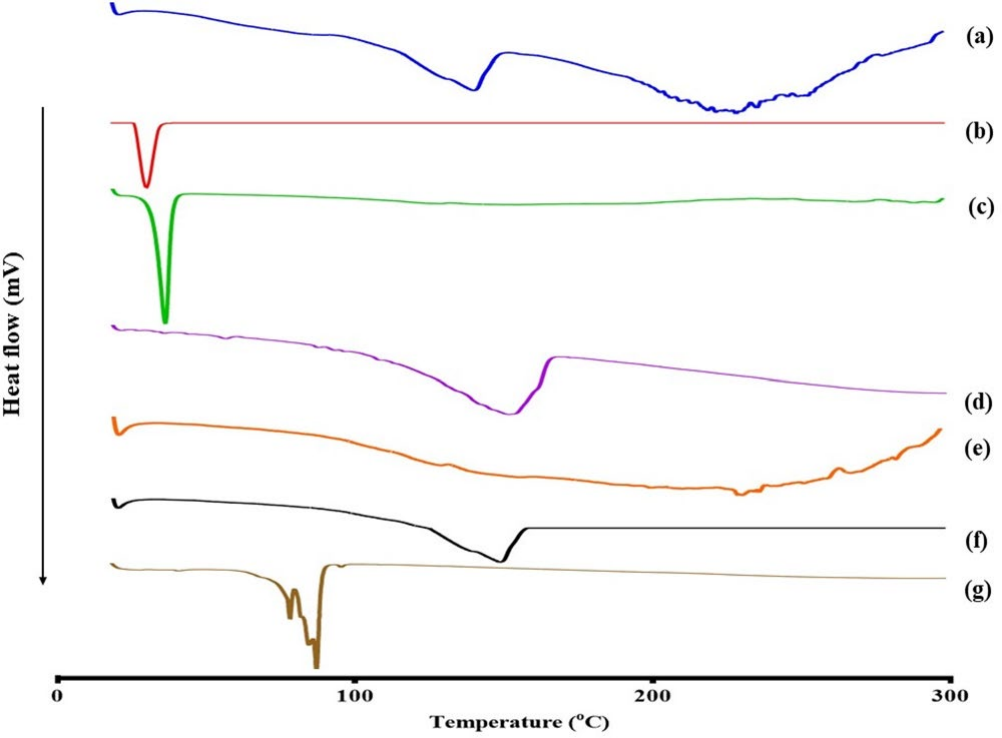
DSC thermograms of (a) pure ATV (b) GMO (c) Kolliphor^®^ P 407 (d) Eugenol (e) Gelucire^®^ 44/14 (f) Physical mixture (g) OEEPC.

DSC thermograms were generated to determine the physicochemical drug-excipient interactions and detect any polymorphic changes. As presented in Figure 3, the DSC thermogram of pure ATV showed an endothermic peak at 154 °C which is corresponding to its melting point and indicating its crystalline state (Yeom et al., 2016). Regarding the physical mixture, the endothermic peak of ATV was preserved indicating the compatibility of the drug with the other cubosomal constituents. On the other hand, the disappearance of the ATV peak from the thermogram of OEEPC indicated the solubilization of ATV in the cubosomal dispersion and its transition from the crystalline to amorphous form (Nasr et al., 2020).

### 3.10. Fourier-transform infrared (FTIR) spectroscopy

**Figure 4.**
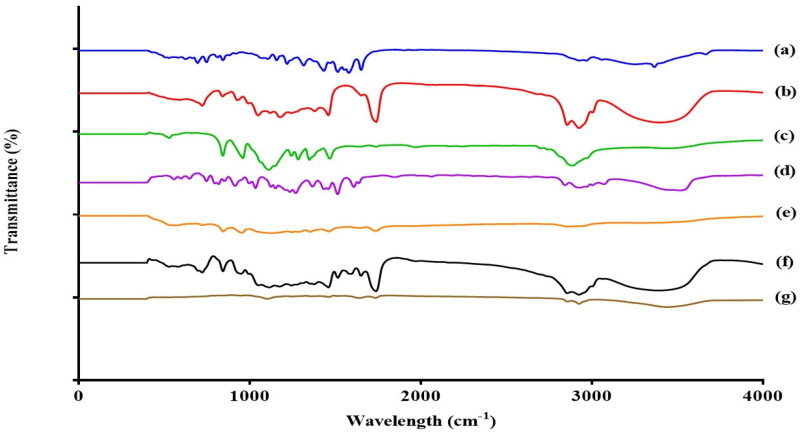
FTIR spectra of (a) pure ATV (b) GMO (c) Kolliphor^®^ P 407 (d) Eugenol (e) Gelucire^®^ 44/14 (f) Physical mixture (g) OEEPC.

FTIR is utilized as a tool to confirm the solubilization of ATV in cubosomal dispersion. As presented in Figure 4, the O-H groups in ATV appeared as two sharp peaks at 3665 cm^−1^ and 3251 cm^−1^, while the characteristic peak of the N-H stretching appeared at 3364 cm^−1^. The stretching of the amide C = O group appeared as a single peak at 1651 cm^−1^, the C-N stretching appeared at 1314 cm^−1^, whereas the aromatic out plane bending appeared at 695 cm^−1^ (Shahraeini et al., 2020). The bands of ATV were preserved in the spectrum of the physical mixture but with lower intensities indicating no interference between the drug and other excipients. The disappearance of the drug bands from the OEEPC spectrum might have indicated its efficient solubilization within the cubosomal matrix.

### 3.11. Stability study

Upon storage, OEEPC was found to preserve its milky appearance with no phase separation or sedimentation on standing. Additionally, there was an insignificant decrease in the values of SE% and ZP and an insignificant increase in the values of PS, PDI, Q_0.5h,_ and Q_8h_ (*p* > 0.05) as shown in Table 3. Upon comparing the release profiles of both fresh and stored OEEPC, it was obvious that both release patterns were almost superimposed, and the obtained results were confirmed by the computed similarity factor (ƒ_2_ = 75). Such findings confirmed the stability of the OEEPC at the specified storage conditions with no remarkable changes. This might be attributed to the synergistic steric stabilization effect imparted by Gelucire^®^ 44/14 and Kolliphor^®^ P 407 and the electrostatic stabilization effect imparted by the high negative charge (Huang et al., 2017; Albash et al., 2021).

**Table 3.** Effect of 30-day storage on the physicochemical properties of the optimized formulation (OEEPC) at 4 – 8˚C. Data are displayed as mean ± SD, (*n* = 3).

Parameter	DC (%)	SE (%)	PS (nm)	PDI	ZP	Q_0.5h_ (%)	Q_8h_ (%)
Fresh OEEPC	109.48 ± 1.07	97.71 ± 0.49	135.20 ± 1.53	0.09 ± 0.006	−28.30 ± 1.84	8.17 ± 2.69	76.99 ± 1.17
Stored OEEPC	109.37 ± 0.92	97.91 ± 0.12	143.20 ± 1.53	0.16 ± 0.01	−26.40 ± 2.37	8.42 ± 2.67	76.53 ± 1.70

**Abbreviations:** OEEPC, optimized eugenol enriched PEGylated cubosomes; DC%, drug content; SE%, solubilization efficiency; PS, particle size; PDI, polydispersity index; ZP, zeta potential; Q_0.5h_, percent of drug released after 0.5 h, and Q_8h_, percent of drug released after 8 h.

### 3.12. Preparation of in-situ gel

In Table 4, different concentrations of Kolliphor^®^ P 407 were tested to select the formulation that can maintain its solution nature at room temperature to be easily injected, yet form a gel once administered into the periodontal pocket for prolonged action, hence reducing the dosing frequency.

**Table 4.** Gelation temperature of ISG formulations containing the optimized formulation (OEEPC). Data are displayed as mean ± SD, (*n* = 3).

Formulations	Kolliphor^®^ P 407 concentration (%w/v)	HA concentration (%w/v)	Gelation temperature (^º^C)
ISG-1	9	0.2	41.00 ± 1.41
ISG-2	9.5	0.2	34.00 ± 0.70
ISG-3	10	0.2	29.25 ± 0.77

**Abbreviations:** ISG, in-situ gel; OEEPC, optimized eugenol enriched PEGylated cubosomes, and HA, hyaluronic acid.

### 3.13. Gelation temperature

**Figure 5.**
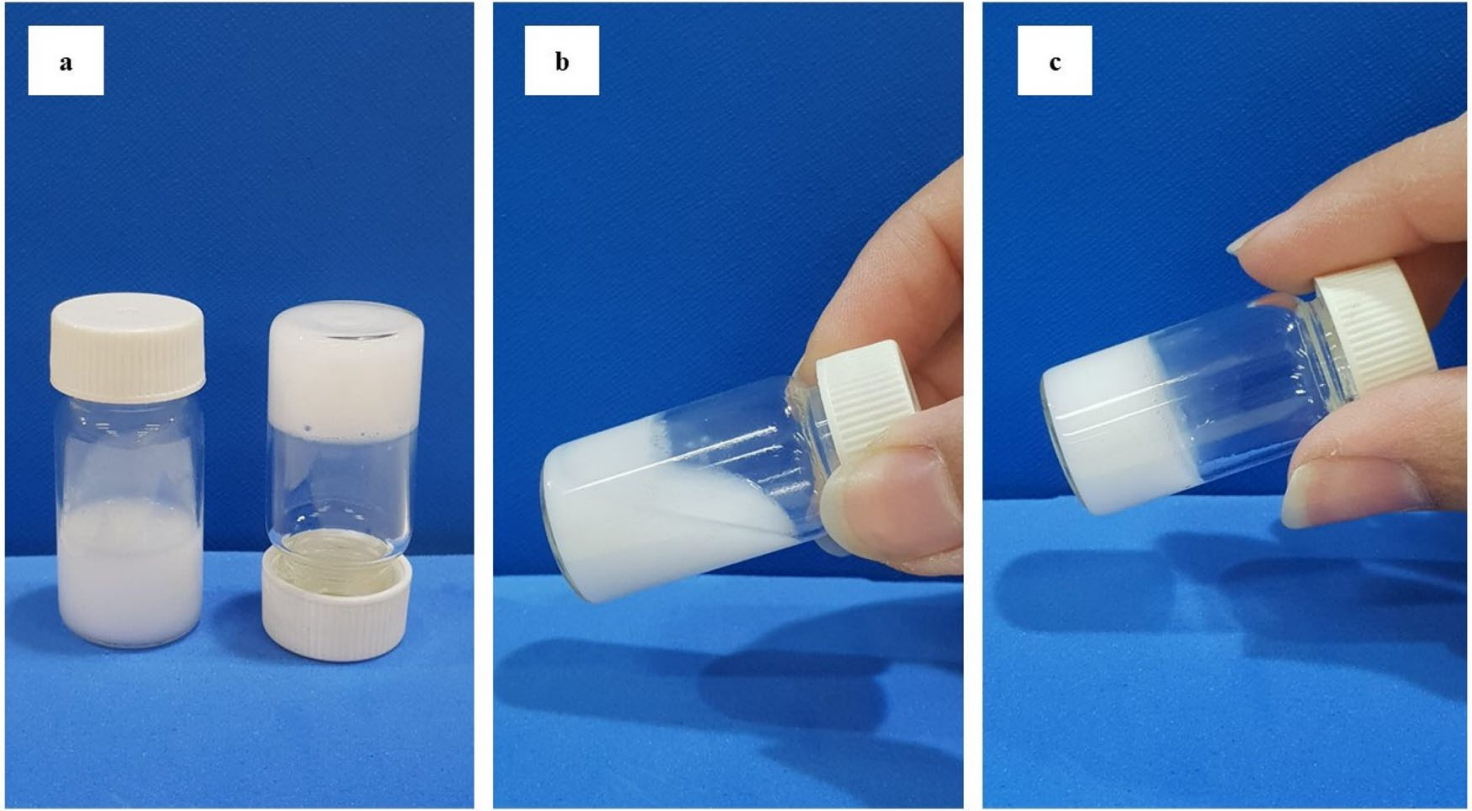
(a) Sol-Gel transition of ISG-2, (b) Sol state at room temperature, (c) Gel state at 34 ± 0.70 °C.

The gelation temperature is an extremely critical parameter for the evaluation of in-situ gel formulations. As shown in Table 4, it was observed that increasing Kolliphor^®^ P 407 concentration in the ISG formulations resulted in a noticeable decrease in the gelation temperature and this result was similar to that observed by Morsi et al. (Morsi et al., 2016). Kolliphor^®^ P 407 is a nonionic triblock copolymer that is comprised of hydrophobic polypropylene oxide (PPO) blocks and hydrophilic polyethylene oxide (PEO) blocks, both contribute to the micellar aggregation phenomenon (Eldeeb et al., 2019). Above critical micelle concentration, the Kolliphor^®^ P 407 molecules arrange to form large micelles with a PPO core and a PEO shell. Micelle formation is known to be easily affected by temperature changes. Elevating the temperature dehydrates the PPO core of the micelles. Afterward, these micelles entangle forming a 3 D network leading to the gelation of the ISG (Morsi et al., 2016). Generally, the temperature of the periodontal pockets ranges from 33.4 to 36.1 °C (Haffajee et al., 1992). In-situ gels with gelation temperature below this range would form a rigid and non-injectable gel. While in-situ gels with gelation temperature above this range would drain easily from the periodontal pocket. So, ISG-2 (9.5% Kolliphor^®^ P 407 and 0.2% HA) with a gelation temperature of 34.00 ± 0.70 °C was selected as the ideal ISG for the intra-pocket delivery of ATV. The sol-to-gel transition behavior of the ISG-2 is represented in Figure 5.

### 3.14. Gelation time

Gelation time is the time taken by the ISG to complete the sol-to-gel transition from the solution state to the gel state at a certain gelation temperature. In the current investigation, it was noticed that the ISG-2 exhibited a gelation time of 46 ± 2.82 sec which was considered ideal for preventing the formulation leakage upon insertion into the periodontal pockets.

### 3.15. Viscosity and rheological behavior measurement

The small surface area of the periodontal pockets necessitates the administration of only a minimal amount of the ISG. So, the formulated ISG should be easily instilled at room temperature and retains its viscosity in the periodontal pockets for better drug localization and retention. The sol-to-gel transition was quantitatively determined by measuring the viscosity of the ISG-2 at room temperature (25 °C) and gelation temperature (34 °C). At 10 rpm, the viscosity of the ISG-2 at 25 °C was 3327.5 cps, while it was doubled to 7024.5 cps at 34 °C indicating gel formation.

**Figure 6.**
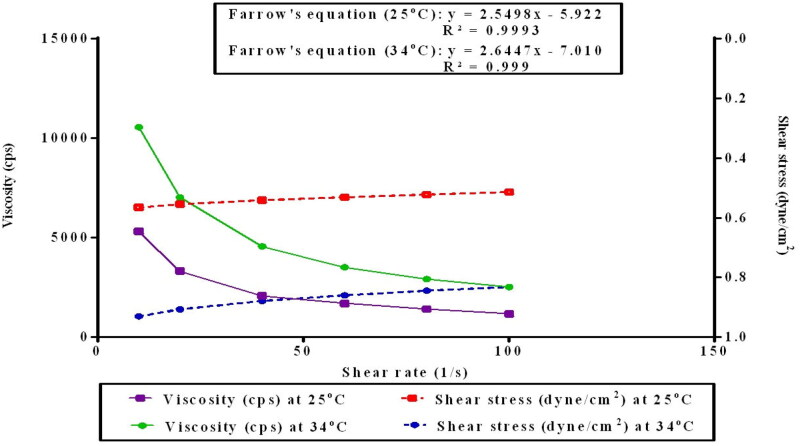
Rheological characterization of ISG-2 (a plot of viscosity and shear stress in relation to shear rate). The measurements were performed at 25 ± 2˚C (■) and 34 ± 2˚C (●), at varying shear speeds (10–50 rpm) with 10 s between every two consecutive speeds.

As shown in Figure 6, the rheological behavior of the ISG-2 at 25 °C and 34 °C exhibited a shear thinning pseudoplastic flow where the viscosity decreased upon increasing the shear rate. This behavior was confirmed using Farrow’s equation where the calculated Farrow’s constant (N) at both temperatures (25 °C and 34 °C) surpassed 1 (*N* = 2.5498 and 2.6447), respectively (Elakkad et al., 2021). Such flow behavior might be attributed to the entanglement of the polymeric molecules forming the ISG-2 at the relaxation condition. Conversely, disentanglement of the polymeric molecules occurred following the exposure to shear stress, permitting the release of the trapped solvent, and decreasing the apparent viscosity. Accordingly, the formulated ISG-2 intended to be applied to the periodontal pockets should preferably follow a pseudoplastic behavior. At 25 °C, the ISG-2 was easily administered due to the shear applied upon its expulsion from the syringe, hence facilitating its flow through the syringe needle. Yet, upon insertion into the periodontal pockets (34 °C), the flow behavior of the ISG-2 would recover rapidly retaining its high viscosity due to the limited exposure to shear stress at the target site. The flow behavior of the ISG-2 was similar to that observed from previously published articles (Bruschi et al., 2007; Srivastava et al., 2016).

### 3.16. Syringeability study

The syringeability of the ISG-2 was qualitatively assessed at room temperature. It appeared to be syringeable and easily injected through a 21-gauge needle syringe, ensuring the ease of administration upon its clinical application.

### 3.17. In-vitro release study of in-situ gel

**Figure 7.**
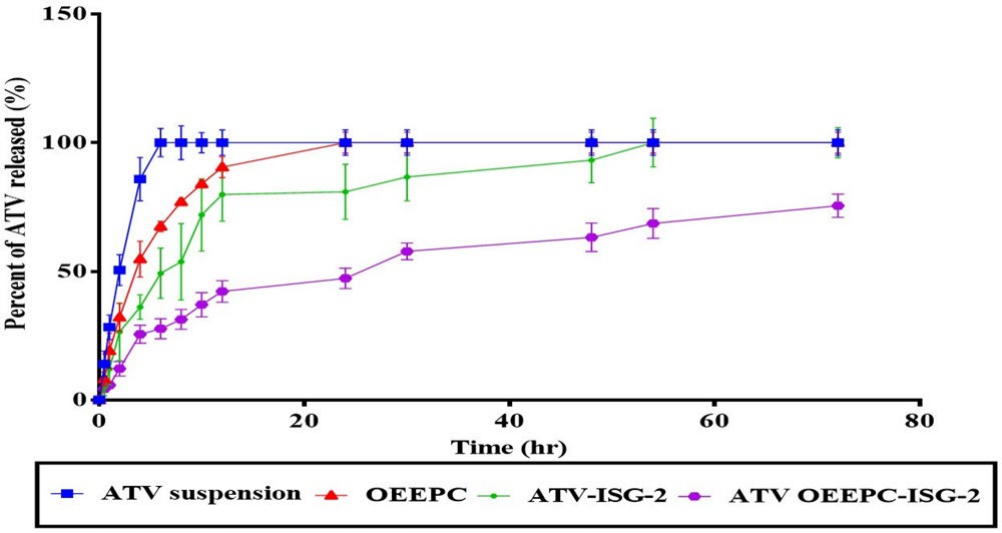
*In-vitro* release of ATV from ATV suspension, OEEPC, ATV loaded ISG-2, and ATV loaded OEEPC – ISG-2.

As shown in Figure 7, 100% of the drug was released from the ATV suspension at 6 h while all the drug was released from the OEEPC after 24 h. This higher rate of ATV release from the drug suspension than that from OEEPC (*p* = 0.077) might be due to the increased binding of the hydrophobic drug to the hydrophobic region of cubosomes which retarded the drug release (Jin et al., 2013). Also, the rate of ATV release from both the OEEPC and ATV loaded ISG-2 was higher than that from the OEEPC loaded ISG-2 (*p* = 0.0001) because the latter is a dual system formed by the inclusion of OEEPC in the in-situ gel which acted as an additional barrier restricting the release of the drug into the dissolution medium. These results are in parallel to that achieved by Mansour et al. who reported that the release of Repaglinide was slower from the ISG than that from the cubosomal dispersion (Mansour et al., 2021). The mathematical modeling of the drug release revealed that the OEEPC and ATV loaded ISG-2 followed the first-order model with the highest r^2^ (0.9985 and 0.9636, respectively) showing that the release of the drug was proportional to the remaining drug concentration in the formulation (Nithya et al., 2018; Chen et al., 2019; Rapalli et al., 2021). While the OEEPC loaded ISG-2 followed Higuchi diffusion model with the highest r^2^ (0.9550) and this result was in accordance with that obtained by Mansour et al. (Mansour et al., 2021). Moreover, the Korsmeyer–Peppas model was applied to detect the mechanism of drug release according to the value of the release exponent (n). The n value of OEEPC was 0.587 (0.45 < *n* < 0.89) which depicted the non-Fickian or anomalous behavior of the drug release which includes diffusion and erosion (Mohsen et al., 2021). While the n value of ATV loaded ISG-2 and OEEPC loaded ISG-2 was 0.366 and 0.424, respectively (*n* ≤ 0.45) indicating a Fickian diffusion mechanism (Gaballa et al., 2020).

### 3.18. Clinical study design

#### 3.18.1. Clinical parameters

##### 3.18.1.1. Probing depth (PD)

**Figure 8.**
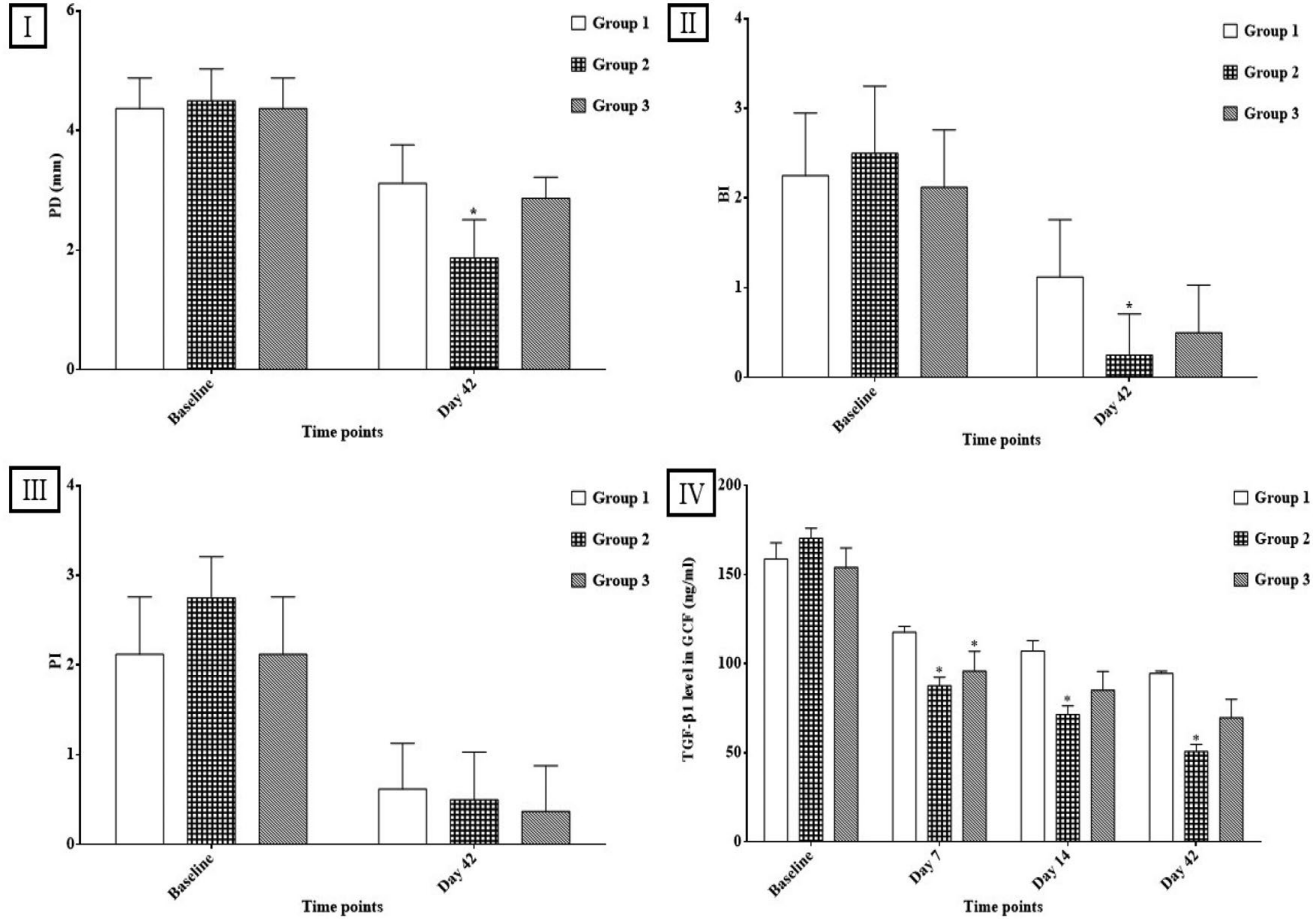
The effects of different treatment groups on (I) PD, (II) BI, (III) PI, and (IV) the concentration of transforming growth factor–β1 (TGF-β1) (ng/ml) in GCF. *p < 0.001 when compared to group 1 (receiving scaling and debridement only)

As presented in Figure 8I, the statistical intra-group comparison at the end of the study showed a significant reduction in the mean PD values (*p* < 0.001) and this was in accordance with a previously published study (Aslroosta et al., 2021). This might be attributed to the decrease in bacterial bioburden as a result of the mechanical removal of plaque and calculus and the meticulous oral hygiene restricted by the patients (Aslroosta et al., 2021). While the statistical inter-group comparison revealed that group 2 showed the highest statistical reduction in PD values with a mean value of 4.50 ± 0.53 at baseline reduced to 1.87 ± 0.64 at the end of the study (*p* < 0.05). Table 5 documents the superiority of group 2 treated with OEEPC loaded ISG-2 offering the most significant percent reduction (58.33%) when compared to the other groups (*p* < 0.01). A probable explanation for this result was the anti-inflammatory and antioxidant actions of ATV and its ability to halt disease progression (Kumari et al., 2017; Tahamtan et al., 2020). Previous literature reported that ATV might decrease the nuclear factor-*ĸ* B ligand, in addition to its ability to scavenge the reactive oxygen species which might result in periodontal tissue destruction (Kumari et al., 2017). Additionally, the formulation of ATV in a dual system (cubosomes and ISG) resulted in a retarded drug release over a suitable period and improved the intimacy of contact leading to a more significant periodontal pocket healing (Jin et al., 2013; Mansour et al., 2021).

**Table 5.** Clinical parameters evaluation in different groups. Data are displayed as mean ± SD, (*n* = 8).

Clinical Parameters	Group 1	Group 2	Group 3
% Reduction in PD	28.57 ± 10.60	58.33 ± 13.61^a^	34.28 ± 12.79^b^
% Reduction in BI	50.00 ± 21.36	90.00 ± 15.43^a^	76.47 ± 25.09
% Reduction in PI	70.58 ± 23.46	81.81 ± 23.14	82.35 ± 23.57

^a^ *p* < 0.01 when compared to group 1.

^b^ *p* < 0.01 when compared to group 2.

**Abbreviations:** PD, probing depth; BI, bleeding index; PI, plaque index, and TGF-β1, transforming growth factor-β1.

##### 3.18.1.2. Bleeding index (BI)

As presented in Figure 8II, the statistical intra-group comparison at the end of the study showed a significant reduction in the mean BI values (*p* = 0.0001) due to scaling and debridement resulting in the resolution of gingival inflammation (Nasra et al., 2017). While the statistical inter-group comparison revealed that both treated groups (2 and 3) with p-values of 0.009 and 0.059, respectively showed a decrease in BI values when compared to group 1 with better results for group 2. Table 5 documents the superiority of group 2 treated with OEEPC loaded ISG-2 offering the most significant percent reduction (90%) when compared to the other groups (*p* < 0.01). This can be referred to the anti-inflammatory effect of ATV in downregulating the production of many pro-inflammatory cytokines (IL-6, IL-8, etc) as reported by Kumari et al. (Kumari et al., 2017). Additionally, previous studies reported that ATV induced a reduction in oxidative stress that was responsible for the destruction of periodontal structure in periodontitis patients (Gumus et al., 2016). It was also able to promote angiogenesis through various mechanisms such as stimulation of vascular endothelial growth factors release and enhancement of the production of nitric oxide (Fam et al., 2003). Moreover, the HA present in the ISG might have played a vital role in the regeneration of periodontal defects and contributed to periodontal wound healing (Dahiya & Kamal, 2013; Fujioka-Kobayashi et al., 2017). Furthermore, the prominent results attained by group 2 treated with ATV loaded OEEPC – ISG-2 might be attributed to the anti-inflammatory effect of eugenol found in cubosomes causing a synergistic reduction in periodontal inflammation (Ahmad et al., 2019).

##### 3.18.1.3. Plaque index (PI)

As presented in Figure 8III, the statistical intra-group comparison at the end of the study showed a significant reduction in the mean PI values (*p* = 0.0001). However, the inter-group comparison revealed a non-significant difference (*p* > 0.05) and this result was similar to that of a previously published study (Gad et al., 2017). This might be due to the supragingival plaque removal along with the meticulous oral hygiene restricted by the patients in the three groups (Gad et al., 2017).

#### 3.18.2. Assessment of transforming growth factor–β1 (TGF-β1)

The mean levels of TGF-β1 at different periods are presented graphically in Figure 8IV. At baseline, it was noticed that group 2 exhibited the highest mean TGF-β1 levels of all groups (170.50 ± 5.62 ng/ml) indicating a high degree of periodontal inflammation. While at day 7, the statistical inter-group comparison showed that both treated groups (2 and 3) had a significant decrease in TGF-β1 levels compared to group 1 (*p* = 0.0001) with a greater reduction in inflammation in group 2 patients (87.66 ± 4.84 ng/ml). These findings proved that the mechanical removal of plaque through scaling and debridement alone did not provide satisfying healing of periodontitis. On the contrary, both formulations had beneficial effects on periodontitis that might be attributed to the anti-inflammatory and immunomodulatory effects of ATV as discussed before (Fam et al., 2003; Tahamtan et al., 2020). Moreover, HA found in both formulations might have accelerated the healing of periodontal lesions through its anti-inflammatory, antioxidant, and wound-healing effects ( Dahiya & Kamal, 2013; Mesa et al., 2002 ). Furthermore, the sol-to-gel transition of these formulations upon administration might have maintained high ATV and HA concentrations in the periodontal pockets.

Whereas on days 14 and 42, the statistical inter-group comparison showed that group 2 had the highest statistically significant decrease in the mean TGF-β1 levels with values (71.61 ± 4.89 ng/ml) and (50.78 ± 3.99 ng/ml), respectively (*p* < 0.001). Group 2 treated with OEEPC loaded ISG-2 proffered an overall 70.21% reduction in the mean TGF-β1 levels throughout the study period when compared to group 1 (40.36%) and group 3 (54.71%). These results demonstrated the supremacy of loading ATV into a dual system (cubosomes and ISG) over a conventional ISG. Given that cubosomes might have played an important role in improving drug absorption and bioavailability owing to the structural resemblance of GMO and the biological lipids forming the periodontal tissues (Mei et al., 2017). In addition to the mucoadhesive nature of cubosomes, the use of PEGylated surfactant (Gelucire^®^ 44/14) might have enhanced the localization of ATV into the periodontal pockets. Furthermore, the high SE% and low PS of the cubosomes guaranteed an ultimate drug loading at the target site with enhanced drug penetration (Mou et al., 2019). Also, as previously discussed, eugenol exerted additional anti-inflammatory and antioxidant effects in improving periodontitis.

## 4. Conclusion

The current study succeeded in establishing an innovative delivery system of atorvastatin calcium (ATV) with promising clinical outcomes in the treatment of periodontal pockets. ATV loaded eugenol enriched PEGylated cubosomes (EEPCs) were fabricated and statistically analyzed and optimized. The optimized formulation (OEEPC) was loaded into a syringeable in-situ gel (ISG) formed of 9.5% Kolliphor^®^ P 407 and 0.2% hyaluronic acid (HA). The conducted clinical study demonstrated that the formulation of ATV in a dual system (cubosomes and ISG) meets the therapeutic dental needs and can be an innovative approach serving as adjunctive therapy to scaling and debridement in the treatment of moderate periodontitis. Moreover, these findings may serve as a roadmap for future plans to develop and execute more extensive clinical investigations on refractory cases.
